# Assessing the Effect of mHealth Interventions in Improving Maternal and Neonatal Care in Low- and Middle-Income Countries: A Systematic Review

**DOI:** 10.1371/journal.pone.0154664

**Published:** 2016-05-04

**Authors:** Stephanie Felicie Victoria Sondaal, Joyce Linda Browne, Mary Amoakoh-Coleman, Alexander Borgstein, Andrea Solnes Miltenburg, Mirjam Verwijs, Kerstin Klipstein-Grobusch

**Affiliations:** 1 Julius Global Health, Julius Center for Health Sciences and Primary Care, University Medical Centre, Utrecht, The Netherlands; 2 School of Public Health, University of Ghana, Legon, Accra, Ghana; 3 Department of Community Medicine, Institute of Health and Society, University of Oslo, Oslo, Norway; 4 Afya Connect(4)Change, Change Lake Zone, Mwanza, Tanzania; 5 International Institute for Communication and Development, The Hague, The Netherlands; 6 Division of Epidemiology and Biostatistics, School of Public Health, Faculty of Health Sciences, University of the Witwatersrand, Johannesburg, South Africa; University of Rochester, UNITED STATES

## Abstract

**Introduction:**

Maternal and neonatal mortality remains high in many low- and middle-income countries (LMIC). Availability and use of mobile phones is increasing rapidly with 90% of persons in developing countries having a mobile-cellular subscription. Mobile health (mHealth) interventions have been proposed as effective solutions to improve maternal and neonatal health. This systematic review assessed the effect of mHealth interventions that support pregnant women during the antenatal, birth and postnatal period in LMIC.

**Methods:**

The review was registered with Prospero (CRD42014010292). Six databases were searched from June 2014–April 2015, accompanied by grey literature search using pre-defined search terms linked to pregnant women in LMIC and mHealth. Quality of articles was assessed with an adapted Cochrane Risk of Bias Tool. Because of heterogeneity in outcomes, settings and study designs a narrative synthesis of quantitative results of intervention studies on maternal outcomes, neonatal outcomes, service utilization, and healthy pregnancy education was conducted. Qualitative and quantitative results were synthesized with a strengths, weaknesses, opportunities, and threats analysis.

**Results:**

In total, 3777 articles were found, of which 27 studies were included: twelve intervention studies and fifteen descriptive studies. mHealth interventions targeted at pregnant women increased maternal and neonatal service utilization shown through increased antenatal care attendance, facility-service utilization, skilled attendance at birth, and vaccination rates. Few articles assessed the effect on maternal or neonatal health outcomes, with inconsistent results.

**Conclusion:**

mHealth interventions may be effective solutions to improve maternal and neonatal service utilization. Further studies assessing mHealth’s impact on maternal and neonatal outcomes are recommended. The emerging trend of strong experimental research designs with randomized controlled trials, combined with feasibility research, government involvement and integration of mHealth interventions into the healthcare system is encouraging and can pave the way to improved decision making on best practice implementation of mHealth interventions.

## Background

The availability and use of mobile phones is increasing rapidly in low- and middle-income countries (LMIC) [1–3]. In 2014, 90% of persons in developing countries have a mobile-cellular subscription (pre-paid and post-paid), 89% in the Asia-Pacific region and 69% in Africa [2]. These countries are responsible for more than 75% of mobile-cellular subscriptions globally [2]. The wide availability of mobile phones and their ease of use have given rise to the field of mobile health (mHealth), in which mobile phones and tablets support medical and public health practice [3–6]. mHealth interventions can be used to provide varying functions: educational information, support, reminders, emergency response, and monitoring [7]. In LMIC this means mHealth could reduce time, distance, and cost of information delivery, and thus overcome issues of inadequate financing, poor access to information, and limited human resources [8]. mHealth interventions are being used for health care strengthening by governments, non-governmental organizations (NGOs), donors, multilateral agencies and corporations in LMIC [3,6].

One of the key-areas addressed by mHealth interventions is the support of pregnant women during the antenatal, birth and postnatal period, in order to tackle high maternal and neonatal mortality [3]. Maternal and neonatal mortality remain high in LMIC despite progress in Millennium Development Goals (MDGs) 4 and 5 [9]. Of the 289,000 maternal deaths in 2013, 286,000 occurred in developing regions [10]. Sub-Saharan Africa (SSA) accounted for 62% of all maternal deaths in 2014 [10]. Similarly, LMIC account for the majority of the 2,612,100 neonatal deaths worldwide [11,12],which is approximately 40% of the deaths of children under five [1].

Between 2011 and 2013, Noordam et al., Tamrat and Kachnowski, and Philbrick published reviews assessing the effectiveness of mHealth interventions targeting maternal and neonatal care [13–15]. Given the relatively emerging field of research and the wide interest in mHealth interventions to improve maternal and neonatal health, a substantial number of studies were published since. In addition, the reviews had quality limitations.

The increased drive to develop and scale-up mHealth interventions, demands availability of robust evidence of the effect [5].Therefore, the main objective of this study was to conduct a systematic review to assess the effect of mHealth interventions targeted at pregnant women to improve maternal and neonatal care in LMIC.

## Methods

### Protocol and registration

This review is part of a larger systematic review which also included mHealth interventions focussed on midwives and health care providers bestowing maternal and neonatal care. It was registered with the PROSPERO review of registry for systematic reviews (CRD42014010292), and is based on the guidelines provided by PRISMA [16](S1 File).

### Eligibility criteria

Studies focussing on the domain of pregnant women during antenatal, labour and postnatal care up to 28 days postpartum in LMIC, and the determinant mHealth were eligible for inclusion. LMIC were defined according to the World Bank Classification [17]. mHealth was defined as a medical and public health practice supported by mobile phones and tablets, making use of text, audio, images, video or coded data in the form of short messaging services (SMS), voice SMS, applications accessible via general packet radio service (GPRS), global positioning system (GPS), third and fourth generation mobile telecommunications, and Bluetooth. mHealth supports the exchange of health related information and provides varying functions: educational information, support, reminders, emergency response, and monitoring [7]. Outcomes were not pre-specified in the search or eligibility criteria given the interest in any outcomes related to our domain and intervention. Studies were excluded when their outcomes did not address outcomes within the antenatal, labour and postnatal period up to 28 days postpartum.

Articles were eligible for inclusion when written in English, Dutch, French, German or Spanish, contained the pre-defined domain and determinant, and were a primary study. Data of results published multiple times were used only once, based on the most comprehensive publication. When an intervention was discussed in more than one article, the study was counted as one, but the outcomes of the different articles shown separately. Articles that included mHealth in a package alongside other non-mHealth interventions, were considered separately. Exclusion criteria included articles not matching the domain and determinant, reports, proceedings, conference abstracts, project protocols and secondary analyses. Interventions relating to the termination of pregnancy were excluded due to the focus on maternity services. Family planning was excluded when it did not start within the post-partum period. Interventions using radio were also excluded as they fell outside the scope of the definition of mHealth. Personal digital assistants were only included when their use in the intervention fit the given definition of mHealth.

### Information sources and search

The systematic literature search was conducted during June 1^st^, 2014 and August 31^st^, 2014 in five electronic bibliographic databases, The Cochrane Library (Cochrane Database of Systematic Reviews), PubMed/MEDLINE, EMBASE, Global Health Library and POPLINE using pre-defined search (Title/Abstract) and indexing terms (MeSH/Emtree) linked to the domains and determinant (see S2 File for search syntax for each database). As many mHealth interventions are not published in the peer-reviewed literature [18], the systematic literature research was complemented by an extensive grey literature search conducted between October 2014 and April 2015. A list of organisations conducting mHealth activities including NGOs, governments, and the WHO working group on mHealth (mTERG) was compiled (S3 File). Their websites were searched for publications fitting the eligibility criteria. Personal contacts (authors from the field MV and ASM, having met through working in the field, or at conferences) were also included and approached. Reference lists of included studies were screened for additional articles that fit the eligibility criteria.

### Study selection

All duplicate articles were removed manually using Endnote (version 11). Screening based on title and abstract was done independently by two reviewers for the database search (SFVS and AB) and by four reviewers for the grey literature search (ASM, MV, SFVS and AB). Any discrepancies between the two reviewers in this process were discussed with the other review team members until consensus was reached and full text was accessed if necessary for further clarification. When access to full-text articles was not available, authors were contacted once. If no reply was received within a month, the study was excluded.

### Data collection process and data items

Data extraction of the database articles was done by one reviewer (SFVS) and four reviewers extracted data of the grey literature articles (ASM, JB, MV and SFVS). None were blinded for journal or author details according to a standardized data extraction form. Other authors double-checked the data extraction for accuracy (AB, KKG and MC). Information was extracted on: study design [19,20], research methods, location/healthcare setting, target population/size, mHealth function (educational, monitoring, reminder, communication and support, and emergency medical response system), form of mHealth (unidirectional text/voice messaging, direct two-way communication, multidirectional text messaging, unidirectional telephone counselling), results per classification (maternal outcomes, neonatal outcomes, maternal and neonatal service utilization, and education), strengths, weaknesses, opportunities and threats of the intervention. Classification of mHealth functions and forms was determined after identification of emerging themes from the results [21]. Where possible, the summary measures risk ratio and odds ratio were used for results. Lack of clarity during the extraction process was resolved by consulting members of the research team (AB, ASM, JB, KKG, MC and MV) until consensus was reached. In case of incomplete data, one attempt was made to contact the corresponding author or organization by e-mail.

### Risk of bias assessment

The intervention articles were assessed for quality according to an adaptation of the Cochrane Risk of Bias Tool for intervention studies [19]. Bias was assessed on the sequence generation, allocation concealment, selection process of the study population, completeness of data (e.g. number of drop-outs), origin of the data (measurements performed by authors or database research), blinding of the researchers or clinicians, the presence of a clear definition of the outcomes that were used, and whether confounders were taken into account. Bias risk was assigned as either one of three levels (low/high/or unclear risk) and taken into consideration in determining the strength of the conclusion as described in the discussion section. The quality assessment criteria are available in the S1 Table. Risk of bias could not be assessed across studies through a funnel plot or Egger’s tests.

### Synthesis of results

After data collection the data was evaluated in order to determine whether a meta-analysis was possible, as originally intended (only reference to the protocol here). Given the heterogeneity in outcomes, settings and study designs, results could not be polled to conduct a meta-analysis. Data synthesis therefore aimed to give a narrative review of mHealth interventions and results. The quantitative results of intervention studies were summarized in an evidence table according to study type: randomized controlled trial (RCT) and non-randomized study (NRCT) [20]. A narrative synthesis of the results per classification was given. The descriptive studies were summarized in a similar evidence table to the intervention studies.

A narrative synthesis of qualitative information was performed with an analysis of the strengths, weaknesses, opportunities, and threats (SWOT) of all the included studies for the domains accessibility, acceptability, and usability. The SWOT analysis was done by one of the reviewers (SFVS, supported by JLB, MV and ASM). The rationale behind conducting a SWOT analysis was the analytical framework that it provides for the identification of internal (strengths and weaknesses) and external factors (opportunities and threats) that influence the effect and possible scale-up of mHealth interventions [18,22].

## Results

**Fig 1:**
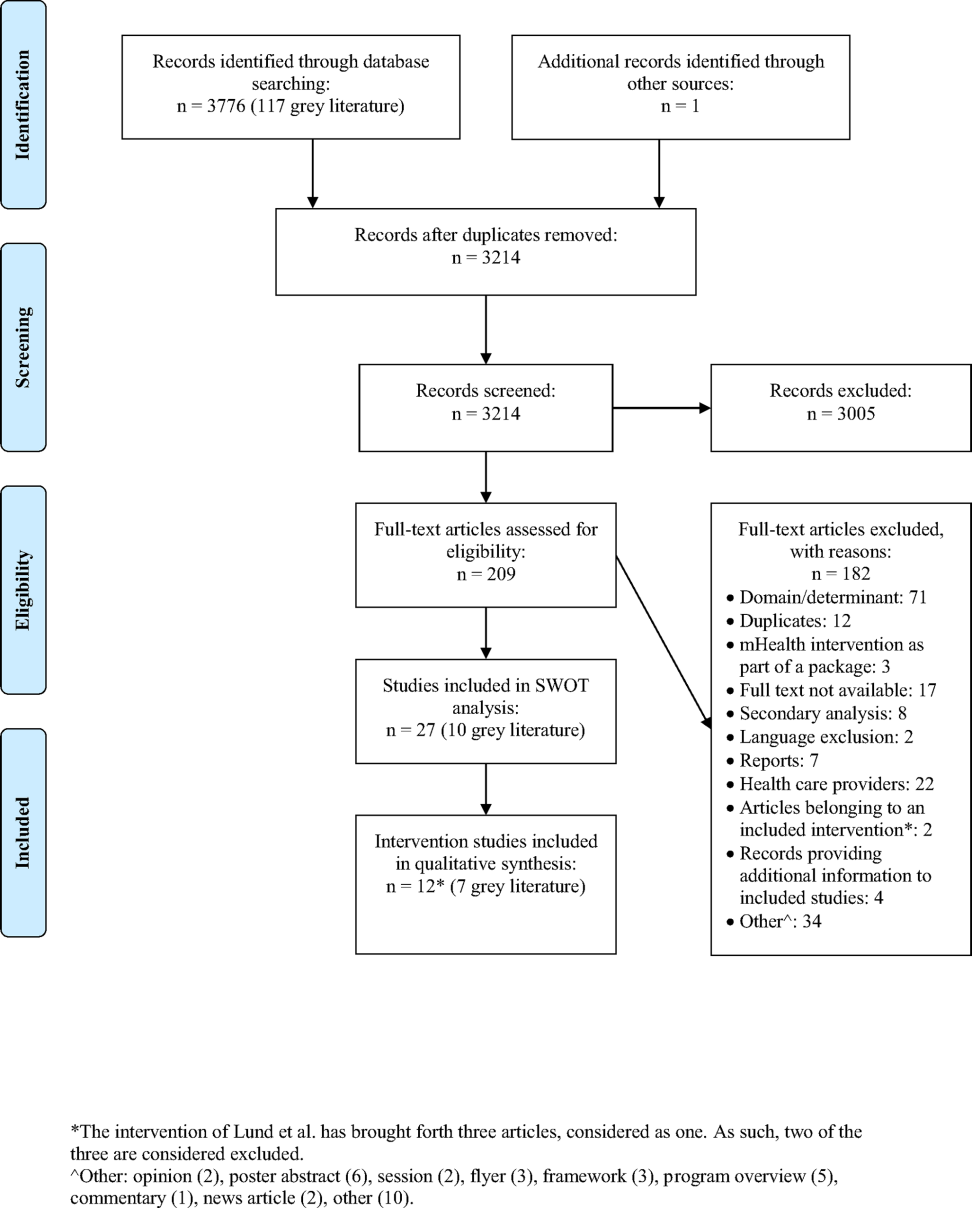
Flow diagram.

**Fig 2:**
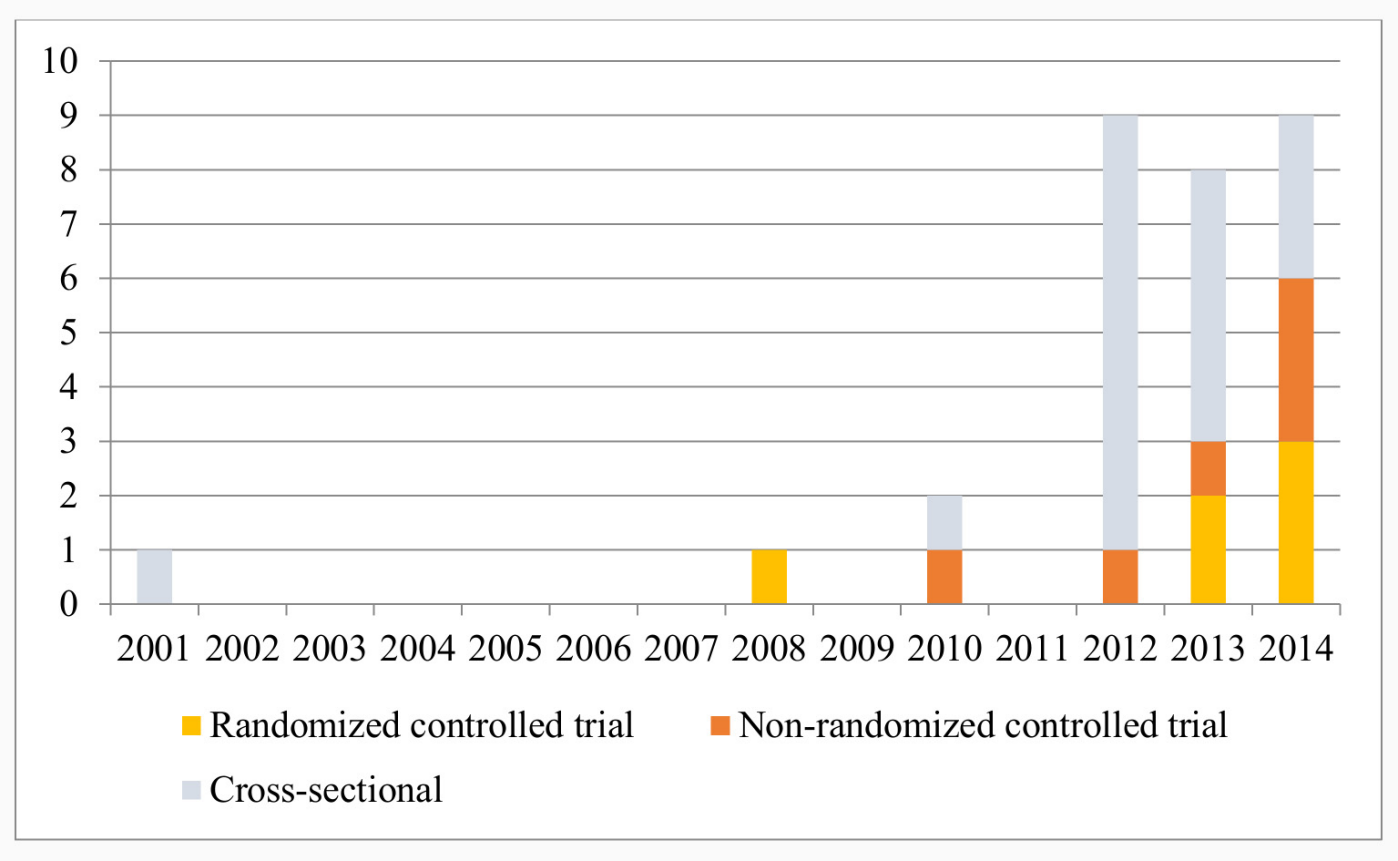
Number of published records of mHealth studies (experimental (RCT and NRCT)) and descriptive) per year.

The search yielded a total of 3214 articles (3659 through database searching and 117 through the grey literature search) after removal of duplicates and 1 article through snowballing (Fig 1). After full-text screening, a total of 29 articles were included. Of these, fourteen were intervention studies and fifteen were descriptive studies. Of the intervention studies, three belonged to one intervention [23–25] and were counted as one intervention study, leaving a total of twelve intervention studies included. Table 1 provides an overview of the scope of the included articles and the number of published records of mHealth studies per year is shown in Fig 2. Tables 2 and 3 give an overview of the characteristics and results of the intervention and descriptive studies respectively. All studies were included for the SWOT analysis (Table 4).

**Table 1:** Overview of scope of the studies (n = 27).

	Intervention studies (n = 12)		Descriptive studies (n = 15)	
Category	Result (n)	Result (%)	Result (n)	Result (%)
**Region**				
Africa	5	42	7	47
Asia	6	50	3	20
Middle-East	1	8	2	13
Europe	0	0	1	7
South-America	0	0	2	13
**Rural/urban**				
Rural	5	42	3	20
Urban	5	42	4	27
Both	1	8	5	33
Unclear	1	8	3	20
**Form of mHealth**				
Unidirectional text (and voice) messaging	6	50	5	33
Direct two-way communication	2	17	4	27
Both unidirectional and direct two-way communication	2	17	1	7
Multidirectional text messaging	1	8	1	7
Unidirectional telephone counselling	1	8	0	0
**mHealth function**^**1**^				
Educational	10	83	9	60
Monitoring	0	0	1	7
Reminder	7	58	6	40
Communication and support	2	17	4	27
Emergency medical response system	4	33	2	13

^1^Several studies used mHealth for multiple functions.

**Table 2:** Characteristics and results of the intervention studies (n = 12).

Study	Study characteristics	mHealth function	Form of mHealth	Maternal outcomes	Neonatal outcomes	Maternal and neonatal service utilization	Education on a healthy pregnancy
**Randomized study design**							
Khorshid et al. 2014	**Study design:** RCT / **Research methods:** Interview and blood sample analysis / **Location:** Iran / **Health care setting:** 20 public health clinics in urban setting / **Target population:** Pregnant women / **Size:** Intervention: 58, Control: 58	*Educational*: on iron supplements (better absorption, side effects, function). / *Reminder*: to take one iron supplement daily.	*Unidirectional text messaging*: 3 reminders and 4 educational health messages every week for 12 weeks	No significant difference was found between groups regarding Hb, Hct and serum ferritin level before and after the intervention. / **High compliance** in the intervention group (94% versus 66% in the control group, *p* = 0.003).	NA	NA	NA
Lau et al. 2014	**Study design:** RCT / **Research methods:** Mixed methods. *Quantitative*: questionnaire. *Qualitative*: FGDs. / **Location:** South Africa / **Health care setting:** Primary health care facility in urban setting / **Target population:** pregnant women / **Size:** Intervention: 102, Control: 104	*Educational*: antenatal health information, staggered according to the week of pregnancy.	*Unidirectional text messaging*: staggered according to the week of pregnancy	NA	NA	NA	No statistical significant difference in score in any of the 9 questions between the intervention and control group. / Intervention group reported fairly healthy behaviours during pregnancy.
Lund et al. 2014	**Study design:** RCT / **Research methods:** Structured questionnaires and recorded patient data / **Location:** Tanzania / **Health care setting:** Primary health care facilities in rural and urban setting / Target population: pregnant women / **Size:** Intervention: 1351, Control: 1286	*Educational*: range of topics related to pregnancy. / *Reminder*: for the next antenatal care visit. / *Emergency medical response system*: access to emergency obstetric care and referral mechanisms through free call voucher system.	*Unidirectional text messaging / Direct two-way communication*: free call voucher system (Wired Mothers)	NA	NA	**Receiving 4 or more ANC visits:** OR 2.39 (95% CI, 1.03–5.55; 44% intervention group, 31% control group). / **Quality of care indicators** were higher for intervention women compared to control women, but not significant.	NA
Lund et al. (1) 2014	**Study design:** RCT / **Research methods:** Structured questionnaires and recorded patient data / **Location:** Tanzania / **Health care setting:** Primary health care facilities in rural and urban setting / Target population: pregnant women / **Size:** Intervention: 1351, Control: 1286	*Educational*: range of topics related to pregnancy. / *Reminder*: for the next antenatal care visit. / *Emergency medical response system*: access to emergency obstetric care and referral mechanisms through free call voucher system.	*Unidirectional text messaging / Direct two-way communication*: free call voucher system (Wired Mothers)	NA	**Stillbirth rate:** 17 per 1000 births in the intervention group, 26 per 1000 births in the control group (OR, 0.65; 95% CI, 0.34–1.24). / **Perinatal mortality:** OR 0.50 (95% CI, 0.27–0.90; perinatal mortality rate (per 1000 births): intervention group: 19, control group: 36. / **Neonatal mortality:** OR 0.79 (95% CI, 0.36–1.74; neonatal mortality rate (per 1000 live births): intervention group: 14, control group: 15.	NA	NA
Lund et al. 2012	**Study design:** RCT / **Research methods:** Structured questionnaires and recorded patient data / **Location:** Tanzania / **Health care setting:** Primary health care facilities in rural and urban setting / Target population: pregnant women / **Size:** Intervention: 1351, Control: 1286	*Educational*: range of topics related to pregnancy. / *Reminder*: for the next antenatal care visit. / *Emergency medical response system*: access to emergency obstetric care and referral mechanisms through free call voucher system.	*Unidirectional text messaging /Direct two-way communication*: free call voucher system (Wired Mothers)	NA	NA	**Delivery with skilled attendance:** intervention group 60%, control group: 47%. Higher effect for urban women: OR, 5.73 (95% CI, 1.51–21.81). / **Increasing socioeconomic class** (completing secondary school or being a sale woman, compared to being a farmer, receiving other forms of education and not owning a mobile phone) was associated with increasing levels of skilled delivery attendance.	NA
Ross et al. 2013	**Study design:** RCT / **Research methods:** Mixed methods. *Quantitative*: questionnaire and depression scale. *Qualitative*: IDI. / **Location:** Thailand / **Health care setting:** Prenatal clinic / Target population: HIV-positive pregnant women / **Size:** Intervention: 20, Control: 20	*Educational*: informational support. / *(Emotional) support*: possibility of calling a registered nurse (and midwife).	*Direct two-way communication*: weekly telephone support	Depressive symptoms amongst participants in the intervention group decreased significantly over time (*p* = 0.125).	NA	NA	NA
Tahir and Al-Sadat 2013	**Study design:** RCT / **Research methods:** Questionnaire / **Location:** Malaysia / **Health care setting:** Public hospital in urban setting / Target population: Pregnant women of a single infant / **Size:** Intervention:179, Control: 178	*Educational*: to encourage exclusive breastfeeding.	*Unidirectional telephone counselling*: on lactation, twice monthly	NA	NA	NA	Exclusive breastfeeding 1^st^ month postpartum: intervention group: 84.3%, control group: 74.7% (unadjusted OR, 1.83; p = 0.042). When adjusted for significant factors, the OR was not significant: 1.63 (95% CI, 0.822–3.22).
Jareethum et al. 2008	**Study design:** RCT / **Research methods:** Questionnaire and recorded patient data / **Location:** Thailand / **Health care setting:** Hospital in urban setting / Target population: Pregnant women / **Size:** Intervention: 32, Control: 29	*Educational*: information and warnings relating to abnormal symptoms during pregnancy. / *Emergency medical response system*: in case of abnormal symptoms, required to consult the doctor.	*Unidirectional text messaging*: twice weekly	**Satisfaction scores** of the antenatal and perinatal period were significantly higher in the study group compared to the control group (9.25 versus 8.00, *p*<0.001; 9.09 versus 7.90, *p* = 0.007 respectively). / **Confidence levels** in the antenatal period were significantly higher amongst the study population (8.91 vs 7.79, *p* = 0.001). They were also higher in the perinatal period but not significant (8.94 vs. 8.38, *p* = 0.074). / **Anxiety levels** were significantly lower in the study population during the antenatal period (2.78 vs 4.93, *p* = 0.002). They were also lower in the perinatal period but not significant (4.78 vs. 5.79, *p* = 0.122). / **No difference in pregnancy outcomes** between groups regarding gestational age at birth and route of delivery.	No difference in pregnancy outcomes between the groups regarding foetal birth weight and preterm delivery.	NA	NA
**Non-randomized study design**							
Datta et al. 2014	**Study design:** Before-and-after study / **Research methods:** Mixed methods. *Quantitative*: questionnaire. *Qualitative*: FGDs. / **Location:** India / **Health care setting:** 6 rural villages / Target population: Residents of the district / **Size:** 120 respondents in 120 households	*Educational*: standard MCH practices.	*Unidirectional text messaging*	NA	NA	NA	Significant increase in respondent's knowledge after the intervention with regards to: (95% CI) no. of TT injections to be received (0.17–0.42); min. no. of iron folic acid tablets to consume (0.21–0.46); min. no. of ANC visits during pregnancy (0.16–0.38); at least two danger signs during pregnancy (0.08–0.31); min. gap between two successive pregnancies (0.11–0.36); when to call a baby as “low birth weight” (0.12–0.35); age up to which to exclusively breastfeed (0.14–0.39); age to initiate complementary feeding (0.03–0.28).
Jalloh-Vos et al.^**a**^ 2014	**Study design:** NRCT (step-wedge approach) / **Research methods:** Mixed methods. *Quantitative*: survey, questionnaire, and data collection. *Qualitative*: SSIs, FGDs, summary info from reports. / **Location:** Sierra Leone / **Health care setting:** Rural / Target population: Pregnant women / **Size:** Wedge 1: 5708, Wedge 2: 2402	*Reminder*: of appointments / *Educational*: health education specific to pregnant women’s situation	*Direct two-way communication*: between healthcare workers themselves, between healthcare workers and pregnant women, between healthcare workers and TBAs	NA	NA	Intervention showed a significant positive net effect on **facility-based service utilization**, for 7 out of 10 of selected indicators: ANC1, ANC4, facility delivery, PNC1, PNC2, PNC3 and newly initiated family planning. / **Increase in facility births and decrease in community births**.	NA
Oyeyemi and Wynn 2014	**Study design:** NRCT / **Research methods:** Retrospective record review / **Location:** Nigeria / **Health care setting:** Health care facilities (general hospital, comprehensive health centres, basic health centres), mainly rural. / Target population: Pregnant women / **Size:** Intervention: 1429, Control: 1801	*Educational*: access to family planning information */ Communication*: communicate free of charge with the healthcare services and among themselves. / *Reminder*: medical appointments. / *Emergency medical response system*: express communication possible in times of an emergency.	*Multidirectional text messaging*: free, CUG cell phone, and participants were assigned to a Health Ranger	No significant difference between the project and the control area (OR = 1; 23 cases of maternal deaths in the project area compared to 29 cases in the control area).	NA	Facility utilization rate: significantly higher in project area than the control area (43.4% vs. 36.7%, *p* = 0.0001), specifically primary healthcare facilities (54.5% vs. 30.6%, *p* = 0.001).	NA
Watkins (Chipatala Cha Pa Foni (CCPF))^**a**^ 2013	**Study design:** Controlled before-and-after study / **Research methods:** Mixed methods. *Quantitative*: questionnaire. Q*ualitative*: FGDs, IDIs, ethnographies, key informant interviews, client exit interviews, rapid facility assessments. / **Location:** Malawi / **Health care setting:** Rural (4 health centre catchment areas). / Target population: Pregnant women, guardians of children under one year of age and women of childbearing age / **Size:** 6479	*Educational*: on appropriate care seeking and health practices / *Reminders*: on appropriate care seeking and health practices / *Emergency medical response system*	*Direct two-way communication*: toll-free case management hotline providing protocol-based health information, advice and referrals. / *Unidirectional text and voice messaging*: personalized tips and reminders service on appropriate care seeking and health practices.	NA	NA	**CCPF had a positive effect on facility-based practices for women:** increase in women attending ANC within the first trimester and a marginally statistically significant increase in PNC check-ups within 2 days after birth. / **CCPF had a negative effect on facility-based services for children:** a substantial reduction in the number of children visiting a health center for fever occurred (almost 60% points).	**The knowledge of many indicators** was already high at baseline, of several indicators knowledge increased in the intervention area and of several others they increased in the control area, as such knowledge did not differ significantly between the two areas. / **CCPF had a positive effect on home-based practices for women and children:** women reported increased use of bed nets during pregnancy (25% points) and breastfeeding a child within one hour after birth (15% points), and children's use of a bednet among children whose mother or caretaker used CCPF increased (25% points). / **The positive effect of knowledge increase** in the intervention area was larger for those who lived at a distance from the health center.
Pathak^**a**^ 2012	**Study design:** Before-and-after study / **Research methods:** Recorded patient data / **Location:** India / **Health care setting:** Urban / Target population: Mothers / **Size:** 509 (of which 263 gave birth)	*Reminder*: vaccinations of their infants in the first year	*Unidirectional text messaging / Direct two-way communication*: if mother had not come with her child two weeks after scheduled vaccination.	NA	NA	Among the 263 mothers, the first dose of BCG/HBV/OPV has a 95% rate, a second dose rate of 98%, and a third dose rate of 100%. Total vaccination rates at baseline were around 60%.	NA
Kaewkungwal et al. 2010	**Study design:** Before-and-after study / **Research methods:** Recorded patient data / **Location:** Thailand / **Health care setting:** 482 households in a rural area. Health care staff in one community hospital and 12 health centres. / Target population: Pregnant women and healthcare providers / **Size:** Pregnant women: 280	*Reminder*: for ANC visits and EPI services (Mother and Child Care Module, MCCM).	*Unidirectional text messaging*	NA	NA	**58.68% come on-time as for their ANC visits** after MCCM implementation compared to 43.79% before (*p*<0.001). / **Sending reminders via text messaging** had an OR for visiting ANC visit on-time of 2.97 (95% CI, 1.60–5.54). / **44.22% came on-time for immunization** after MCCM implementation compared to 34.49% before (*p*<0.001). / **Sending appointment message** increased the odds of receiving EPI on-time by 1.48 (95% CI, 1.09–2.03).	NA

Legend:

^a^: Found through grey literature search, RCT: randomized controlled trial, Hb: haemoglobin, Hct: haematocrit, OR: odds ratio, CI: confidence interval, NRCT: non-randomized controlled trial, HR: hazard ratio, ANC: antenatal care, IDI: in-depth interviews, SSI: semi-structured interviews, FGD: focus group discussion, MDR: maternal death reports, PHUR: peripheral health unit reports, PNC: post-natal care, TBA: traditional birth attendants, MCH: mother and child health, TT: tetanus toxoid, GA: gestational age, CES-D: Centre for Epidemiologic Studies Depression, EBF: exclusive breastfeeding, CUG: Closed-Users' Group, EPI: extended programme on immunization, BCG: Bacillus Calmette-Guérin, HBV: Hepatitis B vaccine, OPV: oral polio vaccine.

**Table 3:** Characteristics and relevant findings of the descriptive studies (n = 15).

Study	Study characteristics	mHealth function	Form of mHealth	Findings
GSMA mHealth^a^ 2014	**Study design:** Cross-sectional / **Purpose of study:** Feasibility / **Research methods:** Face-to-face interviews / **Location:** South Africa / **Health care setting:** National / **Target population:** Pregnant women, mothers and caretakers of children up to the age of 2 / **Size:** 2056	NA	NA	**Most women** are generally "self-aware" about their personal health needs. / **Most women** rely on traditional media and experts for health advice, though a compelling business case can be formulated. / **Including other** services (i.e. job advice) can make service more impactful/relevant among BoP women. / **Only half** of respondents are willing to pay, even though there is a general interest. Women at the BoP are less likely willing to pay compared to non-BoP. / **40% of** target users are aware of existing MNCH messaging services, only half use them.
MAMA South Africa^a^ 2014	**Study design:** Cross-sectional / **Purpose of study:** Reflective evaluation / **Research methods:** NA / **Location:** Developing countries. MAMA is based in South Africa / **Health care setting:** NA / **Target population:** Expected mothers and environment, in low income settings / **Size:** 500.000 subscribers	*Educational*: pre-natal and postpartum care / *Reminder*: ANC visits	*Unidirectional text messaging*: age and stage-based / *Other*: Interactive stage based platform (Mxit), interactive mobile web community platform (Mobisite)	Launched in May 2013. In May 2014 already 350.000 women registered.
BBC Media Action^a^ 2013	**Study design:** Cross-sectional / **Purpose of study:** Feasibility **/ Research methods:** Qualitative: FGs, triad interviews, IDIs / **Location:** Afghanistan / **Health care setting:** Urban and rural areas / **Target population:** New mothers and fathers, pregnant women, mothers-in-law, community health workers / **Size:** Not mentioned	NA	NA	**Mobile phones** are being used increasingly and equally accessible to women as they are to men. For women especially, the phones serve a functional purpose: to keep in contact with their husband and close family. / **Families seek** advice from various sources at the community level: religious leaders, traditional midwives and health clinics, but the ultimate decision-maker is the husband. It is therefore important to direct significant messages to other gatekeepers as well. / **Using mobile** technology for health educational purposes was a well-received concept and seen as cost- and time-efficient compared to visiting a health clinic. / **Some concerns** exist as to the level of literacy needed in order for mobile phones to be used and the costs possibly associated with using such services.
Jennings et al. 2013	**Study design:** Cross-sectional / **Purpose of study:** Preliminary study for the development of a cluster RCT assessing the effectiveness of mobile phones in supporting PMTCT. / **Research methods: Q**ualitative: FGs and IDIs / **Location:** Kenya / **Health care setting:** District hospital in rural area / **Target population:** HIV-positive women enrolled in PMTCT, their male partners, community health workers, and nurses / **Size:** 17, 12, 12 and 4 (respectively)	NA	NA	**Mobile phone** ownership was 47%, 33%, and 67% respectively amongst HIV-positive women, their male partners and community health workers and 88%, 83% and 83% respectively had experience with receiving SMS. 35% of the women and 50% of the men shared their mobile phone, compared to 17% of the community health workers. Using mobile phones for health purposes is mostly done using voice calls. / **Perceived benefits** were: ability to request antiretroviral drugs via phone, refer fellow HIV-positive women to a community health worker, quick notification of appointments, rescheduling or the status of drugs and supplies saving time and money. / **Common disadvantages** were: lack of money for buying airtime and charging phones, delays in communication, privacy not always guaranteed, uncertainty of whether complex messages can be sent using mobile communication, difficulty in verifying that a recommended task is followed up. / **All participants** indicated preferring a platform whereby SMS and phone call applications were customized for different purposes: information that is brief and relatively confidential, the opportunity for discussion, and checking whether a message was received well. / **Gender-tailored SMS** were considered as motivating for male involvement in PMTCT and the communication between partners.
MAMA Bangladesh^a^ 2013	**Study design:** Cross-sectional / **Purpose of study:** Formative / **Research methods:** Mixed methods: data collection, SIs, phone surveys, field observation, FGDs. / **Location:** Bangladesh / **Health care setting:** Rural and urban areas (five divisions of Bangladesh) / **Target population:** Pregnant women, mothers, gatekeepers. / **Size:** 349, 575, and 479 respectively.	*Educational*: prenatal and postpartum care / *Reminder*: ANC visits	*Unidirectional text messaging*: staggered according to age and time of pregnancy / *Unidirectional voice messaging*: interactive voice response (IVR)	**IVR is** more popular than SMS messages. / **Assisted registration** is more effective. / **Training and** monitoring CHWs is important for ensuring success. / **Assisting in** registration supports longer exposure to receiving SMS/IVR. / **Registered people** were satisfied with services. / **Satisfied with** services till first birthday of child. / **Behaviour change** is reported.
MAMA Tanzania^a^ 2013	**Study design:** Cross-sectional / **Purpose of study:** Reflective evaluation / **Research methods:** Not specified. / **Location:** Tanzania / **Health care setting:** Rural and urban areas / **Target population:** Pregnant women, their supporters (partner, family, friends), health professionals / **Size:** Not specified.	*Educational*: healthy pregnancy, prenatal and postpartum care / *Reminder*: ANC visits	*Unidirectional text messaging*: stage based	**In 2013**, 1.000 health professionals were oriented about the campaign (Wazazi Nipendeni) and there were 180.000 active subscribers for services. / **Pregnant women** attending ANC visits requested more information related to specific SMS messages about danger signs and nutrition.
Cormick et al. 2012	**Study design:** Cross-sectional / **Purpose of study:** Feasibility / **Research methods:** Mixed methods: data collection and questionnaires / **Location:** Argentina / **Health care setting:** Community health centres and public hospitals in urban areas / **Target population:** Pregnant women. / **Size:** 146	NA	NA	**Mobile phones** were used by 136 of the 146 women (93.2%). Only 6 (4.1%) also used the internet on their cell phones. / **140 of** the 146 (96%) indicated wanting to receive informational text messages about prenatal care. 133 (91%) would also like to receive information postpartum. The time at which varied greatly. / **The topics** most women preferred included: prenatal dietary information (90%), infant dietary information (91%), activities to avoid during pregnancy (92%), when to call a doctor during pregnancy (91%), lactation counselling (91%), infant skin care (95%).
Dean et al. 2012	**Study design:** Cross-sectional / **Purpose of study:** Post-analysis of utilisation of a pilot intervention / **Research methods:** Mixed methods: data collection and follow-up interviews. / **Location:** South-Africa / **Health care setting:** Antenatal clinics in urban townships / **Target population:** HIV-positive pregnant women. / **Size:** 12	*Educational*: provide relevant therapeutic and educational discussion. / *Support*: Connect a small group around a shared experience.	*Multidirectional text messaging*: within a group including participants and health care providers	**In total**, 1018 individual messages were sent over a period of 12 weeks. Most (247) were sent during week 4 when all participants had been enrolled. Participants sent an average of 16 messages per enrolled, technology-problem-free week. / **SMS content** concerned medical information (51%: 32% regarding HIV/PMTCT and 19% regarding health and pregnancy), and psychosocial content (49%: 23% general interaction, 15% relationships and 11% other personal issues). / **Overall participants** were satisfied, felt that their knowledge on HIV had increased and felt more social connectedness despite lack of physical contact. / **The anonymity** and remote access of the intervention enable stigma and certain logistical challenges (lack of time, lack of confidentiality) to be overcome, as well as cater for diverse personalities. / **Phones were** simple to use, messages were clear and easily kept confidential. / **Rapid troubleshooting** and maintenance, as well as providing the SMS messages at inexpensive wholesale prices, are necessary factors to enable feasibility of the project at a larger scale. This would also overcome the issue of intermittent absence of individuals and passive participants sending few messages.
Diallo et al. 2012	**Study design:** Cross-sectional / **Purpose of study:** Formative / **Research methods:** Mixed methods: data collection and interviews. / **Location:** Burkina Faso / **Health care setting:** Health care centres and maternity wards in urban setting. / **Target population:** Mothers of children between 0–5 years / **Size:** 210 (30 groups of 7 mothers)	*Reminder*: vaccination of newborns	NA	**142/210 (68%)** own a mobile phone, of which 115/142 (81%) are able to read an SMS. / **Of the** 27 mothers who could not read an SMS, 18 (66%) could understand a voice SMS in the language Dioula, and 7 (26%) in the language Moré/Peuhle/French. / **100% of** mothers who have a mobile were willing to give their number to the health centre to be given a reminder about vaccination sessions for their child. / **Of the** 68 mothers who do not have their own mobile phone, 9 (13.2%) do not have anyone in the family living in the same household who can receive an SMS. / **56/59 (95%)** of family members of mothers who do not have their own mobile phone are capable of reading an SMS. / **In total** 198/210 (94.3%) of mothers could directly or indirectly through a family member be called to a vaccination session by written or voice SMS.
Jankovic et al. 2012	**Study design:** Cross-sectional / **Purpose of study:** Post-analysis of utilisation / **Research methods:** Questionnaire / **Location:** Serbia / **Health care setting:** Facebook page accessible to pregnant women / **Target population:** Pregnant women / **Size:** 239	*Educational*: principles of drug use in pregnancy, health facilities offering counselling for drug use during pregnancy.	*Facebook page*: accessed via personal computers and smart phones. Provided educational information in a unidirectional manner.	**Of the** 293 registered ‘friends’, 93 women (39%) responded to the questionnaire. / **Of the** 93 respondents, 50 (53.8%) indicated taking medication(s) during their current pregnancy. And of these 50, 42 were assessed by two clinical pharmacology specialists as using one or more drugs improperly. / **None of** the study participants that were given the advice to contact an official facility, followed up on this advice.
JiVitA^a^ 2012	**Study design:** Cross-sectional / **Purpose of study:** Post-analysis of utilization for a pilot study nested within a RCT / **Research methods:** Data collection / **Location:** Bangladesh / **Health care setting:** Rural (19 unions of Gaidbandha and Rangpur Districts) / **Target population:** Pregnant women and newborns / **Size:** 500 and >16,000 births	*Emergency medical response system*: labor notification system to ensure the presence of nurses at birth to collect placentas and cord blood / *Communication and support*: mobile birth notification allowing newborns to be reached for the provision of a vitamin A dose	*Direct two-way communication*	**337/611 of** the total number of "near miss" events used a mobile phone. / **The phone** was used to call the provider (72%), request medical advice (57%), arrange transport (33%) and request financial aid (72%). / **Mobile phone** was used in 89% of deliveries. / **In 68%** of the cases a mobile phone was used, the team arrived prior to placental expulsion. / **Average 15** hours from birth the newborn was reached; *80% of births were reached within 8 hours.
Kuo et al. 2012	**Study design:** Cross-sectional / **Purpose of study:** Post-analysis of utilisation. / **Research methods:** App development and user evaluation / **Location:** China (Taiwan) / **Health care setting:** Medical centre in urban area / **Target population:** Postpartum patients / **Size:** 78	*Educational*: Q&A items for the top six most frequently asked categories. / *Monitoring*: baby’s health status, growth, vaccination status. / *Emergency medical response system*: hot-line button for a direct call to a nurse. / *Reminder*: vaccination.	*Direct two-way communication*:: application providing newborn baby care support	**All participants** had a cell phone, used their phone to make phone calls (100%). 77% used it to send short messages. 36% used a smartphone. / **The app** was ranked highest (on a 5-point scale) in terms ‘ease of learning’ (4.14), followed by usefulness (4.07), acceptance (4.05), the ease of use (3.98), satisfaction (3.86), and trust (3.74). / **84% indicated** willingness to pay up to US$6 a month for the app. / **84% agreed** the app could be used to answer their questions, and 81% regarded the app as being able to meet their needs.
MOTECH/ Grameen^a^ 2012	**Study design:** Cross-sectional / **Purpose of study:** Reflective process evaluation. / **Research methods:** Embedded a qualitative study. / **Location:** Ghana / **Health care setting:** Rural in Upper Eastern Region / **Target population:** Pregnant women and their families / **Size:** Not specified.	*Educational / Reminder*	*Unidirectional text and voice messaging*	**Can result** in behaviour change. / **Increase in** demand needs can put strain on health system resources. / **Women experienced** increased support from husbands and family members after they heard the messages.
Osman et al. 2010	**Study design:** Cross-sectional / **Purpose of study:** Post-analysis of utilisation. / **Research methods:** Analysis of call patterns and content. / **Location:** Lebanon / **Health care setting:** Postpartum wards, both rural and urban. / **Target population:** Healthy first-time mothers / **Size:** 353	*Educational / Support*: emotional	*Direct two-way communication*: telephone hotline accessible 24 hours a day during the first 4 months after delivery	**Of the** 353 women enrolled, 84 (24%) used the hotline. Of these 84, 50 (60%) called more than once, and all were satisfied with the hotline and willing to encourage other women to use it. / **In total**, 312 calls were received. Most calls were made by the mother of the infant (89.1%). / **7.7% of** the calls were followed up upon and 18.6% of the calls were referred to a physician. / **In total** 570 questions were asked, 139 regarding maternal topics (66% concerned breastfeeding) and 377 regarding infant topics (60% concerned routine care, 23% concerned fussiness and crying). / **Utilization is** highest in the first month postpartum, with breastfeeding questions being highest immediately after delivery, infant fussiness questions are highest in the first 3 weeks, and infant care questions are highest in the first 3–4 weeks postpartum. / **The service** does not add a significant workload to the work of the nurses.
Parrilla-Rodríguez et al. 2001	**Study design:** Cross-sectional / **Purpose of study:** Analysis of utilisation. / **Research methods:** Descriptive analysis / **Location:** Puerto Rico / **Health care setting:** Breastfeeding clinic, reaching pregnant women in urban and rural areas / **Target population:** Mothers breastfeeding / **Size:** Not specified	*Educational support*: orientation / *Referral*: to the breastfeeding specialist physician or to the clinic breastfeeding support group or classes.	*Direct two-way communication*: telephone hotline to a breastfeeding counsellor	**78.7% were** breastfeeding fully or giving their baby breast milk only, 21.3% were partially breastfeeding or using formulas. The majority of the calls were made during the baby’s first two months of age (34.7% below one month, 62.8% at 2 months or less). / **Most of** the calls came from the greater San Juan metropolitan area (33.8%), indicating the need for service to be toll free in order to extend its impact. / **Most callers** were referred to the hotline by friends or family members (64.2%). Physicians, hospitals, and other health care providers referred in 13.8% of the time. / **Mothers breastfeeding** fully and those breastfeeding partially called for different reasons: significantly more calls were made by the full breastfeeding group regarding breastfeeding products (19.0%, *p* = 0.02), storage and management of breast milk (11.7%, *p* = 0.0001), medication use (11.7%, *p* = 0.009); significantly more calls were made by the partial breastfeeding group regarding breast feeding position problems or problems with latch-on (8.5%, *p* = 0.01), problems with engorgement (6.9%, *p* = 0.04), breast refusal (6.2%, *p* = 0.001), and relactation (2.3%, *p* = 0.02). / **Most of** the women received orientation (93.3% in the full breastfeeding group and 83.2% in the partial breastfeeding group). Referrals to the breastfeeding specialist were done amongst 12.5% of the full breastfeeding group and 41.1% of the partial breastfeeding group. Referrals to the clinic breastfeeding support groups or classes were 14.5% and 12.6% respectively.

Legend:

^a^: Found through grey literature search, BoP: bottom of pyramid, MNCH: maternal, neonatal and child health, RCT: randomized controlled trial, FGD: focus group discussion, IDI in-depth interview, CSBA: community based skilled providers, TBA: traditional birth attendant, MAMA: Mobile Alliance for Maternal Action, ANC: antenatal care visit, NA: not applicable, SI: structured interview, PMTCT: prevention of mother-to-child transmission, HIV: human immunodeficiency virus, SMS: short messaging service, CHW: community health worker.

**Table 4:** SWOT analysis of the included intervention and descriptive studies (n = 27).

Factors	Internal factors		External factors	
	Strengths	Weaknesses	Opportunities	Threats
**Accessibility**	Providing information in lay terms [23–29] (D: [30])	Lack of mobile phone ownership and/or sharing of phone with partner [23–25,28,37] (G: [34,35,38–41])	Toll free mobile communication [23–25,28] or pay according to income (D: [30,31]) (G: [35])	Need for electricity for charging [23–25] (D: [31]) (G: [38])
	Low costs for user and implementer [23–25,28] (D: [30,31]) (G: [32])	High costs could limit widespread use [23–25,28] (D: [30,31]) (G: [40–42])	Using voice SMS to overcome illiteracy [23–25] (G: [34,35])	Need for a functioning network [23–25] (D: [31]) (G: [38,39,41])
	Developed using locally-based software development expertise [23–25,33]	Illiteracy with text-based messages [23–25,28] (G: [32,35,38,39,41,42])	Increase reach to include women without mobile phone access by including women groups, traditional birth attendants or other figures at the community level [23–25] (G: [38,42])	Distribution of phones may be necessary [37] (G:[34])
	Voice SMS available for those who are illiterate [23–25] (G: [34,35])	Accessibility lower amongst rural women or women of a lower socio-economic status [23–25] (G: [35,38,39,43])	Feasibility study done prior to implementation informs optimization of access (especially for those at the bottom of the pyramid) [44] (G: [38])	Rapid troubleshooting and maintenance needs to be available (D: [30])
	Information available in different (local) languages [29] (G: [36])	Absence of local fonts [26]	Use of incentive schemes to increase recruitment (G: [42])	Those at the bottom of the pyramid with a lack of dispensable income are not willing/able to pay for mHealth intervention (G: [41,42])
	Can penetrate rural areas [33] (G: [35])	Dependent on technical competency of user (G: [40])		(Cultural) aspects that influence access to intervention (women's empowerment, decision making, confidentiality, the perception of difficulty of subscribing) (G: [38,41,42])
		Low mobile literacy (G: [32,40])		
**Acceptance**	Regarded as supportive by pregnant women [23–25,27,44] (D: [30,45]) (G: [34,38])	Recipient fatigue when too many are sent [26] (G: [35,42])	Integrated into existing healthcare system [23–25] (G: [32,34–36,39,42])	Lack of privacy from family members [44] (G: [38])
	Overcomes issue of lack of time amongst pregnant women [29,44] (D [30]) (G: [34,38,41])		Involvement of the government to create supportive environments aligning with health guidelines (G: [36,39,42])	Hesitation to participate because face-to-face interaction is regarded an important aspect of care [46]
	Accessible when convenient to the user [26,29] (G:[34,35])		Feasibility studies prior to implementation can identify gaps and needs [44] (G: [38])	External factors that limit women from following health guidelines (eg. breastfeeding exclusively up till a certain age, when women are also expected to return to work) [46]
	Facilitates quicker response in cases of emergency [37] (G: [38])		Combination of mHealth forms [46]	
	Can decrease the number of visits through mobile phone consultations (G: [34,38])		Schedule message to avoid frustration from unpredictable messages (G: [35])	
	Personalization of messages and linking information to experiences (G: [40,42])		mHealth can support governments’ effort to register pregnant women (G: [36])	
	Overcomes issue of difficulty with transportation [44]			
	Anonymity and remote access through mobile phones can help overcome stigma of HIV/AIDS (D: [30])			
**Usability**	Mobile interventions are flexible, i.e. can be staggered according to period in pregnancy [23–25,27,44]	Uncertainty whether information of message is received correctly [26] (D: [31]) (G: [36])	Combination of mHealth forms allowing different needs to be addressed [38,46]	Privacy is not always guaranteed [44] (D: [31])
	Simple mobile phone technology that is easy to use [23–25,33] (D: [30])	Uncertainty whether message is received (G: [36])	Question-answer system can provide more interaction when SMS messaging is used (G: [39])	Dependent on donor funding, sustainability (G: [39])
	Developing the intervention locally facilitates implementation as it builds on pre-existing knowledge of mobile phone use [23–25]	Voice messages can be missed and are difficult to store for future reference (in comparison to SMS) (G: [34])	Target audience represents a significant group (i.e. all pregnant women) which offers opportunities for advertising/revenue to be generated to make it sustainable (G: [42])	
	Text limits prevent lengthy messages being sent [26]	Text limits require skills to design useful health messages fitting the limit (G: [34])		
		Durability of phones is not always sufficient (G: [34])		

**Legend:** Accessibility: What factors make an intervention accessible to a pregnant woman?; Acceptance: What factors make that the pregnant women enjoy/like/accept the intervention; Usability: What factors influence the usage of an intervention?; D: descriptive, indicating that the source(s) of the point belong(s) to the descriptive studies or studies; G: grey literature, indicating that the source(s) of the belong(s) to the grey literature studies.

### Overall risk of bias assessment of intervention studies

**Fig 3:**
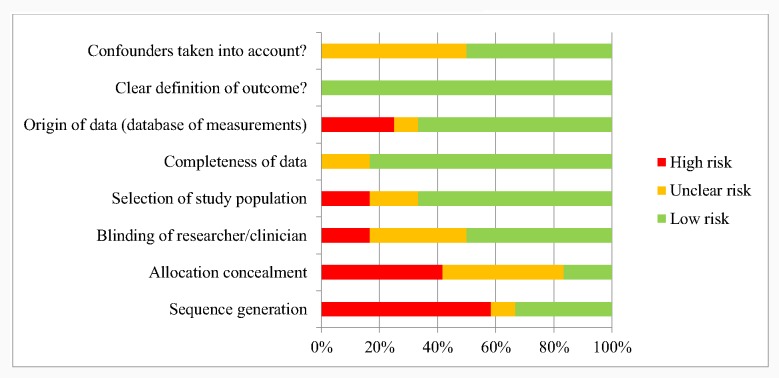
Summary of the overall risk assessment.

A summary of the overall risk assessment is shown in Fig 3 and the quality assessment of the intervention studies are shown in Table 5 with the detailed version available in S2 Table. Intervention articles generally performed well in their risk of bias for the selection of study population (66% low risk), completeness of data (83% low risk), clear definition of outcome (100% low risk) and confounders (50% low risk, with the remainder unclear). A number of studies displayed high risk of bias in sequence generation (58%), allocation concealment (41%), or origin of data (25%).

**Table 5:** Quality assessment of intervention studies (n = 12).

Study	Sequence generation	Allocation concealment	Blinding of researcher/ clinician	Selection of study population	Completeness of data	Origin of data (database of measurements)	Clear definition of outcome?	Confounders taken into account?
**Randomized controlled trial**								
2014 Korshid et al.	**Low risk**	**Unclear risk**	**Unclear risk**	**Low risk**	**Low risk**	**Low risk**	**Low risk**	**Low risk**
2014 Lau et al.	**High risk**	**High risk**	**Unclear risk**	**High risk**	**Low risk**	**Unclear risk**	**Low risk**	**Low risk**
2014 (1), 2014 (2) and 2012 Lund et al.	**Low risk**	**Low risk**	**Low risk**	**Low risk**	**Low risk**	**Low risk**	**Low risk**	**Low risk**
2013 Ross et al.	**High risk**	**High risk**	**Low risk**	**High risk**	**Low risk**	**Low risk**	**Low risk**	**Low risk**
2013 Tahir and Al-Sadat	**Low risk**	**Low risk**	**Low risk**	**Low risk**	**Low risk**	**Low risk**	**Low risk**	**Low risk**
2008 Jareethum et al.	**Low risk**	**Unclear risk**	**Unclear risk**	**Low risk**	**Low risk**	**Low risk**	**Low risk**	**Unclear risk**
**Non-randomized studies**								
2014 Datta et al.	**High risk**	**High risk**	**High risk**	**Low risk**	**Unclear risk**	**Low risk**	**Low risk**	**Unclear risk**
2014 Jalloh-Vos et al.	**High risk**	**Unclear risk**	**Low risk**	**Low risk**	**Low risk**	**Low risk**	**Low risk**	**Unclear risk**
2014 Oyeyemi and Wynn	**High risk**	**High risk**	**Low risk**	**Low risk**	**Low risk**	**High risk**	**Low risk**	**Unclear risk**
2013 Watkins et al.	**High risk**	**Unclear risk**	**Low risk**	**Unclear risk**	**Low risk**	**High risk**	**Low risk**	**Unclear risk**
2012 Pathak	**Unclear risk**	**Unclear risk**	**Unclear risk**	**Unclear risk**	**Unclear risk**	**Low risk**	**Low risk**	**Unclear risk**
2010 Kaewkungwal et al.	**High risk**	**High risk**	**High risk**	**Low risk**	**Low risk**	**High risk**	**Low risk**	**Low risk**

Of the RCTs, Lund et al. and Tahir and Al-Sadat had a low risk for all items [23–25]. Khorshid et al. and Jareethum et al. had an unclear risk regarding allocation concealment and the blinding of the researcher. The latter also had an unclear risk concerning confounders [27,28]. Lau et al. and Ross et al. had high risks of bias in their randomization and selection of study population [29,44]. The studies with a non-randomized study design generally lost quality with regards to sequence generation and allocation concealment and the selection of the study population. Oyeyemi and Wynn, Watkins et al. and Kaewkungwal et al. displayed a high risk in the origin of data [33,37,47]. The study of Pathak had an unclear risk for many items [32]. An unclear risk was often found for the item of confounders [26,32,37,38,47].

### Narrative synthesis of quantitative results

#### Maternal and neonatal service utilization

All studies addressing maternal and neonatal service utilization showed significant increases.

For maternal service utilization, several studies showed positive effects on antenatal care (ANC) attendance. The Wired-Mothers intervention of Lund et al. more than doubled the odds of a woman receiving four or more ANC visits (OR 2.39, 95% CI 1.03 to 5.55) [24]. The pre-post intervention study in Thailand of Kaewkungwal et al. also showed higher ANC attendance rates after reminders were sent via text messaging (ANC visits: OR 2.97, 95% CI 1.60 to 5.54) [33]. The Chipatala Cha Pa Foni program in Malawi by Watkins et al. combined a toll free case management hotline and unidirectional text and voice messaging to provide protocol-based health information and advice on appropriate care seeking, health practices, referrals and reminders. They found both increased ANC and postnatal care attendance (PNC) [47]. In Sierra Leone, direct two-way communication was set up amongst healthcare workers, between healthcare workers and pregnant women, and between healthcare workers and traditional birth attendants, to improve maternal and newborn health service utilization. The intervention showed a positive net effect on facility-based service utilization for the following indicators: first and fourth ANC visit (0.7 and 11.3%-points, facility delivery (8.2%-points), and first, second and third PNC visit (10.1, 10.6 and 14.9%-points) [38]. The effect decreased or became negative, however, when the chiefdom containing the district headquarter town (which is relatively urban and has relatively better and a great variety of services available) was controlled for [38]. Oyeyemi and Wynn found a significantly higher facility utilization rate within the area in Nigeria taking part in a mHealth intervention (43.4% versus 36.7%, *p* = 0.0001) [37]. They defined facility utilization rate as the number of deliveries in a particular health facility to the number of ANC registrations in that same facility [37].

Skilled attendance at birth was increased in the study by Lund et al. (60% in the intervention group compared to 47% in the control group) [25], especially for women in an urban area (OR 5.73, 95% CI 1.51 to 22.81) [25].

Two studies in Thailand addressed the effect of mHealth interventions on the emotional aspects of pregnancy. Jareethum et al. observed significantly higher satisfaction scores in the antenatal and perinatal period and high confidence scores and low anxiety levels when educational text messages were sent twice per week [27]. The study of Ross et al. showed a significant decrease in depressive symptoms amongst the HIV-positive pregnant women in the intervention group which received educational and emotional support via telephone [44].

Regarding neonatal service utilization, the pre-post intervention study of Kaewkungwal et al. showed that reminders via text messaging resulted in a higher services-on-time rates of the extended programme on immunization (EPI) (OR 1.48, 95% CI 1.09 to 2.03) [33]. Similar results were observed by Pathak, in a vaccination project in India with unidirectional text messaging to remind mothers to take their newborn for vaccination. The pilot showed a high success rate: 95% rate of the first dose of BCG/HBV/OPV (second dose rate of 98% and third dose rate of 100%) compared to 60% in total at baseline [32].

#### Maternal outcomes

No studies reported on maternal mortality or severe acute maternal morbidity. Only Oyeyemi and Wynn reported on cases of maternal deaths. They compared the effect of distributing Closed-Users’Group phones to pregnant women and health workers through which they could communicate with each other and among themselves for free in one area to another area in Nigeria and found no significant difference between the two regarding cases of maternal deaths [37].

The study of Khorshid et al. in Iran assessed the effect of educational text messages highlighting the importance of iron supplementation during pregnancy on compliance and anaemia. Results showed significantly higher compliance in the intervention group, assessed according to the number of supplements returned unused by the end of the study. However, no effect on anaemia was found, as there were no significant differences in haemoglobin, haematocrit and ferritin levels[28].

Jareethum et al. found no differences in gestational age at delivery and mode of delivery between pregnant women receiving two educational text messages per week and pregnant women who did not [27].

#### Neonatal outcomes

Lund et al. observed a significant effect on perinatal mortality in their study conducted in Zanzibar. Their Wired Mothers intervention combined unidirectional text messaging and direct two-way communication in a free call voucher system to provide education on pregnancy, reminders for antenatal care visits and an emergency medical response system. They found a significant decrease in the perinatal mortality rate of 50% (OR 0.50, 95% CI 0.27 to 0.90) [25]. The total perinatal mortality rate based on stillbirth and neonatal mortality was 27 per 1000 births, 19 per 1000 births in the intervention group compared to 36 per 1000 births in the control group [25]. Jareethum et al. who assessed the effect of two educational text messages sent weekly in Thailand, found no differences for infant birth weight and preterm delivery [27].

#### Education about a healthy pregnancy

The studies assessing antenatal health knowledge showed varying results. Lau et al. found no statistical difference in antenatal health knowledge assessed by nine questions between the pregnant women receiving educational text messages and those who did not receive messages [29]. The SMS were, however, mentioned as the main source of antenatal health knowledge by participants (98%) [29]. Datta et al. did find a significant increase in respondent’s knowledge after the intervention on several maternal and neonatal topics in their pre-post intervention study [26]. Watkins et al. in Malawi showed that their participant’s baseline knowledge indicator was high at baseline and did not differ significantly between women residing in the intervention and control catchment area, except for women living at a greater distance from the health centre [47]. They observed a positive effect in home-based practices: including use of bed nets during pregnancy and for their children (25% increase for both) and the number of children breastfed within one hour after birth (15% increase).

Tahir and Al-Sadat and Jiang et al. both looked into the effect of unidirectional telephone counselling providing education on breast- and infant feeding practices, in order to encourage exclusive breastfeeding. Tahir and Al-Sadat found that 84.3% of the women in the intervention group breastfed exclusively one month postpartum, compared to 74.7% in the control group with a significant odds ratio of 1.83. However, when adjusted for significant factors relating to exclusive breastfeeding, the odds ratio was no longer significant (OR 1.63, 95% CI 0.82 to 3.22) [46]. Jiang et al. found median durations of exclusive breastfeeding and exclusive breastfeeding rates to be higher in the intervention group (median durations: 11.4 weeks compared to 8.9 weeks (p<0.001); exclusive breastfeeding rates: 15.1% in the intervention compared to 6.3% (adjusted OR 2.67; 95% CI 1.45 to 4.91)) in the control group. They also observed a decreased risk of stopping exclusive breastfeeding amongst the intervention group at six months (OR 0.80, 95% CI 0.66 to 0.97) [48].

### SWOT analysis

The results of the SWOT analysis are presented in Table 4. Accessibility of mHealth interventions was enhanced when information in lay-terms [23–30] and in different (local) languages was provided [29,36], and was developed using locally-based software [23–25,33]. The latter enables troubleshooting to be dealt with more easily. Important weaknesses of described mHealth interventions were the lack of mobile phone ownership [23–25,28,34,35,37–41], high costs [23–25,28,30,31,40–42], illiteracy [23–25,28,32,35,38,39,41,42] and low accessibility to mobile phones amongst rural women [23–25,35,38,39,43]. These weaknesses highlight groups which may not have equal access to mHealth interventions, such as those with a lower socio-economic status, those illiterate and those residing in rural areas out of network coverage zones. As such, opportunities for mHealth are toll-free mobile communication [23–25,28] or paying according to income [30,31,35], voice SMS to target illiteracy [23–25,34,35] and using (women) groups at the community level to reach those women with no access to mobile phones [23–25,38,42]. Important threats that need to be tackled are the need for electricity to charge [23–25,31,38] and the need for a functioning network [23–25,31,38,39,41].

Acceptance was highest when the interventions were able to help overcome and were supportive of issues pregnant women faced [23–25,27,30,34,38,44,45], such as lack of time to travel to the clinic [29,30,34,38,41,44], lack of transport means [44], and difficulty in receiving emergency care [37,38]. The convenience of choosing when to access the information given was also appreciated [26,29,34,35]. A threat to acceptance was recipient fatigue when too many messages were sent [26,35,42], and the importance of face-to-face interaction within maternal care [46]. The importance of privacy could be an issue when the mobile phone is shared amongst family members [38,44]. It also makes pregnant women dependent on others for accessing the intervention. Opportunities to increase the acceptance of mHealth interventions are integration into health care [23–25,32,34–36,39,42], the use of existing health guidelines and close collaboration with government [36,39,42], implementation after a thorough feasibility study had been conducted [38,44], and combining various mHealth forms [46].

Strengths of mHealth interventions on usability included the ease of use [23–25,30,33], flexibility in use and adaptability to the time in pregnancy [23–25,27,44], and development within the local context [23–25]. Weaknesses of mHealth interventions included the uncertainty whether a text is received [26,31,36] and the limited length of messages [34]. A combination of the different forms of mHealth, such as educational text messaging and direct two-way communication for emergencies, provide an opportunity for future interventions [38,46].

## Discussion

This systematic reviewed suggests that mHealth interventions targeted at pregnant women can increase antenatal and postnatal care attendance, facility-based deliveries, skilled attendance at birth, and vaccination rates. No consistent effects of mHealth interventions on maternal and neonatal health outcomes were observed, though single studies describe benefits regarding reduced perinatal mortality and improved breastfeeding practices. Other studied health outcomes such as anaemia, gestational age at delivery, mode of delivery, neonatal birth weight, preterm delivery, stillbirth and neonatal mortality were not significantly affected by mHealth interventions. Inconclusive findings were observed on the effect of mHealth on health knowledge of pregnant women.

The absence of a consistent translation of improved attendance on the continuum of maternal and neonatal health services were also observed in previous reviews [13–15,18], and may be due to the quality of the evidence with moderate risk of bias across studies, especially with non-randomized study designs. Another factor may be substandard care provided at facilities. In fact, mHealth has been proposed as catalyst to identify those areas where strengthening is needed [40]. To explore the impact of mHealth on health outcomes in the future, studies on mHealth interventions should address health endpoints as primary or secondary outcomes and more rigorous study designs are needed that consider the gold standard components to determine an intervention’s effect, i.e. a RCT either on individual or on cluster level. The need for rigorous study designs is amplified by the numerous actors involved in mHealth activities in maternal health. As the grey literature search illustrates, community based and non-governmental organizations are very active in this field. Increased attention to ensure such interventions and activities are evaluated and results disseminated will increase the evidence base, and provide solid evidence on which governments and institutions can base decisions whether to implement mHealth interventions.

mHealth interventions can be implemented in isolation, at several levels within the health system simultaneously, or combined with other inter-sectorial improvements such as infrastructure and capacity of (human) resources [8,13,40,49–51]. mHealth interventions that were combined with non-mHealth interventions showed positive results. Huq et al. set up a toll-free mobile communication network that allowed direct two-way communication for support and emergency situations between pregnant women and community based skilled providers (CSBA), and between CSBAs and medical specialists [52,53]. This was combined with strengthening of the health system, CSBA training, and community support group formation. Mothers perceived the communication with CSBAs as beneficial, improving access of services and overcoming issues of long distances. It also led to more prompt referrals when there were maternal complications and CSBAs could not manage. Mobile communication made it possible to inform pregnant women of the appropriate facility to seek care, decreasing delay in management. Flax et al. showed that breastfeeding learning sessions reinforced through educational text and voice messages sent to participants phones, along with songs and dramas created by participants themselves at micro-credit meetings, improved breastfeeding practices in the neonatal period (initiating breastfeeding within 1 hour of delivery (OR 2.60, 95% CI 1.60–4.10), giving only colostrum or breast milk in the first 3 days of life (OR 2.60, 95% CI 1.40–5.00)) [54]. For the transition towards comprehensive mHealth programs featuring alongside other investments, we recommend greater efforts to collaborate, bring forward and share the evidence of (mHealth) activities.

A strength of this review is that it is, to the best of our knowledge, the first comprehensive review which assesses systematically the evidence on mHealth interventions for maternal and neonatal care, and provides an evaluation of the quality. To allow for a comprehensive overview that considered the activities published outside of the peer-reviewed domain, we also included grey literature and descriptive studies. Combining all studies in a SWOT analysis provided rich data about local needs and context, the optimization in the process of mHealth intervention design and implementation, and what is needed to achieve improved outcomes. Although mHealth interventions should be adapted to local contexts [15], the SWOT analysis showed that several features are considered important across interventions. On the level of technology, the use of simple mobile phone technology and locally developed interventions facilitate implementation by building on pre-existing knowledge of mobile phone use [23–25,30,33]. Access is increased by providing information in lay terms, making information available in different (local) languages, maintaining low costs or toll free mobile communication, and providing voice SMS for those illiterate (Table 4) [23–31,34–36]. The latter is important, as our systematic review did find that inequities arise in who can access mHealth interventions and its potential benefits or not, depending on phone ownership, literacy, rural or urban residency and socio-economic status [23–25,28,32,34,35,37–43]. In addition, almost all RCTs were conducted in urban areas, highlighting the importance of being aware of the possible selection mechanisms that “technology-driven tools and strategies tend to have built-in” which can affect those at the bottom of the pyramid [38].

Including the grey literature allowed us as well to observe the importance of improving collaboration between academic institutions and implementing organizations, as both can learn from each other. This may also address the incongruence between mHealth activity reports in the academic literature and those reported from the field. The quality improvement that NGO-academic collaboration can offer is illustrated by the Wired Mothers project’s RCT with a low risk of bias [23–25]. This also enhances transparency, as the Wired Mothers project is one of the two publicly registered trials (NCT01821222) [23–25]. Lastly, it can result in the definition of a common language and framework when mHealth interventions and evaluations are discussed, across stakeholders and disciplines [4].

A limitation of the systematic review is that a meta-analysis was not feasible based on the data obtained from the include articles. A further limitation is the domain of this systematic review, maternal and neonatal care in LMIC, which resulted in the exclusion of several interventions of potential interest for a comprehensive mHealth intervention along the continuum of maternal, neonatal and child health [15]. These included interventions aimed at family planning [55,56], programs for immunization beyond 28 days [57,58], retinopathy of immaturity [59], and breastfeeding practices [48]. We also did not consider in this review mHealth interventions targeted at health professionals and those conducted in high-income countries. [51,60–67]. Burden of morbidity and mortality is greatest in LMIC, and it is in these countries where mHealth is currently receiving great interest as an innovative method to contribute towards the achievement of Millennium Development Goal 4 and 5, respectively Sustainable Development Goal 3.

Improved communication within the global health community, between NGOs, governments, donors, and other stakeholders, will also help to identify priorities mHealth could and should address [15], and what the position is in relation to the health system and other technological innovations in the domain of eHealth [68]. Feasibility studies and government involvement can help in identifying these priorities and aid and integration of mHealth interventions into the existing healthcare system [18], as well as guiding frameworks and cost-effectiveness assessments.

## Conclusions

mHealth interventions targeted at pregnant women can be an effective solution to increase service utilization to improve maternal and neonatal outcomes. The emerging trend of strong experimental research designs, feasibility research, government involvement and integration of mHealth into the healthcare system is encouraging and can pave the way to improved decision making how best to implement mHealth interventions.

## Supporting Information

S1 File PRISMA 2009 Checklist. (DOC)

S2 File Full search strategy. (DOCX)

S3 File List of organizations included in the grey literature search. (DOCX)

S1 Table Adapted quality assessment tool. (DOCX)

S2 Table Quality assessment for individual articles. (DOCX)
