# Supplementary material for: Assessing the Effect of mHealth Interventions in Improving Maternal and Neonatal Care in Low- and Middle-Income Countries: A Systematic Review
Source: PLoS One. 2016 May 4;11(5):e0154664. doi: 10.1371/journal.pone.0154664 (PMC4856298; doi:10.1371/journal.pone.0154664)
Supplement: S3 File — (DOCX) [file pone.0154664.s003.docx]

**S2 File. List of organizations included in the grey literature search**

Aga Khan

BBC Media Action

Brac - Click Diagnosis

Cell-Life

CommCare

Concern Worldwide

D Tree

Every Woman Every Child

FHI360

Grameen - MOTECH

GSMA (Groupe Speciale Mobile Association)

IICD (International Institute for Communication and Development)

Jhpiego (John Hopkins Program for International Education in Gynaecology and Obstetrics)

KIT (Koninklijk Instituut voor de Tropen)

MAMA (Mobile Alliance for Maternal Action)

Marie Stopes

Medic Mobile

mHealth Alliance

MHTF (Maternal Health Task Force)

Mother2Mother

NEEDS (Network for Enterprise Enhancement and Development Support)

Pathfinder

Pharmaccess

Philani

Partners in Health/Compañeros En Salud

Plan International

Text2Change

Unicef

USAID (United States Agency for International Development)

Vodacom

WHO (World Health Organization)

World Bank

World Vision
