# Supplementary material for: Assessing the Effect of mHealth Interventions in Improving Maternal and Neonatal Care in Low- and Middle-Income Countries: A Systematic Review
Source: PLoS One. 2016 May 4;11(5):e0154664. doi: 10.1371/journal.pone.0154664 (PMC4856298; doi:10.1371/journal.pone.0154664)
Supplement: S1 Table — (DOCX) [file pone.0154664.s004.docx]

| **Risk category** | **Low risk** | **High risk** | **Unclear risk** |
| --- | --- | --- | --- |
| **Sequence generation: (randomisation)** | The investigators describe a random component in the sequence generation process. | The investigators describe a non-random component in the sequence generation process. Usually, the description would involve some systematic, non-random approach. Other non-random approaches happen much less frequently than the systematic approaches mentioned above and tend to be obvious. They usually involve judgement or some method of non-random categorization of participants. | Insufficient information to permit judgment of ‘Low risk’ or ‘High risk’. |
| **Allocation concealment: (randomisation)** | Participants and investigators enrolling participants could not foresee assignment because one of the following, or an equivalent method, was used to conceal allocation: Central allocation (including telephone, web-based, and pharmacy-controlled, randomization); Sequentially numbered drug containers of identical appearance; Sequentially numbered, opaque, sealed envelopes. | Participants or investigators enrolling participants could possibly foresee assignments and thus introduce selection bias, such as allocation based on: Using an open random allocation schedule (e.g. a list of random numbers); Assignment envelopes were used without appropriate safeguards (e.g. if envelopes were unsealed or non-opaque or not sequentially numbered); Alternation or rotation; Date of birth; Case record number; Any other explicitly unconcealed procedure. | Insufficient information to permit judgment of ‘Low risk’ or ‘High risk’. |
| **Blinding: (detection bias)** | No blinding or incomplete blinding, but the review authors judge that the outcome is not likely to be influenced by lack of blinding. | No blinding or incomplete blinding, and the outcome is likely to be influenced by lack of blinding. | Insufficient information to permit judgment of ‘Low risk’ or ‘High risk’. |
|  | Blinding of participants and key study personnel ensured, and unlikely that the blinding could have been broken. | Blinding of key study participants and personnel attempted, but likely that the blinding could have been broken, and the outcome is likely to be influenced by lack of blinding. | The study did not address this outcome. |
| **Selection of study population: (selection bias)**    ***Case-control specific:***      ***Cohort specific:*** | The individuals selected to participate are representative of the target population. | The individuals selected to participate are somewhat likely/not likely to be representative of the target population. | Not described whether individuals selected to participate in the study are likely to be representative of the target population. |
|  | The investigators describe a random component in the sequence generation process. | The investigators describe a non-random component in the sequence generation process. | Insufficient information about the sequence generation process to permit judgement of ‘Yes’ or ‘No’. |
|  | Case definition is adequate with independent validation. | Case definition is adequate, e.g. record linkage or based on self-reports. | No description of the case definition. |
|  | Consecutive or obviously representative series of cases. | Potential for selection biases. | Representativeness of cases not stated. |
|  | Selection of controls occurred from community controls with no history of disease. | Controls are hospital controls. | There is no description of the controls. |
|  | Representativeness of the exposed cohort is truly representative of the average …(describe) in the community. | Representativeness of the exposed cohort is somewhat representative of the average …(describe) in the community. | There is no description of the derivation of the cohort. |
|  | The non-exposed cohort is drawn from the same community as the exposed cohort. | The non-exposed cohort is drawn from a different source. | There is no description of the derivation of the non-exposed cohort. |
| **Completeness: (attrition bias)**              ***Case-control specific:*** | No missing outcome data. | Reason for missing outcome data likely to be related to true outcome, with either imbalance in numbers or reasons for missing data across intervention groups. | Insufficient reporting of attrition/exclusions to permit judgement of ‘Low risk’ or ‘High risk’ (e.g. number randomized not stated, no reasons for missing data provided). |
|  | Reasons for missing outcome data unlikely to be related to true outcome (for survival data, censoring unlikely to be introducing bias). | For dichotomous outcome data, the proportion of missing outcomes compared with observed event risk enough to induce clinically relevant bias in intervention effect estimate. | The study did not address this outcome. |
|  | Missing outcome data balanced in numbers across intervention groups, with similar reasons for missing data across groups. | For continuous outcome data, plausible effect size (difference in means or standardized difference in means) among missing outcomes enough to induce clinically relevant bias in observed effect size. |  |
|  | For dichotomous outcome data, the proportion of missing outcomes compared with observed event risk not enough to have a clinically relevant impact on the intervention effect estimate. | ‘As-treated’ analysis done with substantial departure of the intervention received from that assigned at randomization. |  |
|  | For continuous outcome data, plausible effect size (difference in means or standardized difference in means) among missing outcomes not enough to have a clinically relevant impact on observed effect size. | Potentially inappropriate application of simple imputation. |  |
|  | Missing data have been imputed using appropriate methods. | Lost to follow up is mentioned but there is a large size variation between the patient and control group. |  |
|  | Lost to follow up is mentioned and is comparable within the patient as well as the control group. |  |  |
|  | Non-response rate was same rate for both groups. | Non respondents described. | Non-response rate different and no designation. |
| **Origin:**  ***Cohort specific:*** | Self-measurements or data was gathered by adequate personnel (midwife, research assistant etc.). | Data from database (not collected by researches themselves). | Article does not describe where data came from. |
|  | Assessment of outcome occurred through independent blind assessment and/or record linkage. | Assessment of outcome occurred through self-report. | No description of assessment of outcome. |
| **Definition of outcome:** | Article gives adequate definitions of outcome measurements. | Article does not give adequate definitions of outcome measurements. | Article does not give adequate definitions for ALL outcome measurements. |
| **Confounders:** | Article states that confounders were taken into account and define the confounders. | Article states that confounders were not taken into account. | Article only states that confounders were taken into account, but no specific confounders are given. |
|  |  |  | Article does not state that confounders are taken into account. |
