# Supplementary material for: Assessing the Effect of mHealth Interventions in Improving Maternal and Neonatal Care in Low- and Middle-Income Countries: A Systematic Review
Source: PLoS One. 2016 May 4;11(5):e0154664. doi: 10.1371/journal.pone.0154664 (PMC4856298; doi:10.1371/journal.pone.0154664)
Supplement: S2 Table — (DOCX) [file pone.0154664.s005.docx]

**S2 Table. Quality assessment for individual articles**

| **Study** | **Sequence generation** | **Allocation concealment** | **Blinding of researcher/clinician** | **Selection of study population** | **Completeness of data** | **Origin of data (database of measurements)** | **Clear definition of outcome?** | **Confounders taken into account?** |
| --- | --- | --- | --- | --- | --- | --- | --- | --- |
| **Randomized study design** | | | | | | | | |
| 2014  Korshid et al. | Random number table generated by spreadsheet.  **Low risk** | Not clearly stated.  **Unclear risk** | Not clearly stated.  **Unclear risk** | Randomized.  **Low risk** | No missing outcome data.  Loss to follow-up mentioned and comparable.  **Low risk** | Self-measurements.  **Low risk** | Yes.  **Low risk** | Successful randomisation.  **Low risk** |
| 2014  Lau et al. | Alternately assigned into intervention and control groups.  **High risk** | Allocation based on alternation.  **High risk** | Insufficient information to determine whether low or high risk.  **Unclear risk** | Non-random component in the sequence generation process. The "focus group might have only consisted of very motivated individuals who were happy with the SMS campaign"  **High risk** | No missing outcome data.  Loss to follow-up mentioned and comparable.  **Low risk** | Use of questionnaires testing knowledge is low risk, whereas the outcome regarding health behaviours is self-reported and of high risk.  **Unclear risk** | Yes.  **Low risk** | Despite non-random component in sequence generation, baseline characteristics are not significantly different.  **Low risk** |
| 2014 (1), 2014 (2) and 2012  Lund et al. | 24 eligible clusters were randomized using simple random allocation.  **Low risk** | 24 eligible clusters were randomized using simple random allocation.  **Low risk** | “Neither study participants nor clinic staff were masked because of the nature of the intervention requiring overt participation.”^21^. Judge that outcome is not influenced.  **Low risk** | Randomized and large sample size.  **Low risk** | No missing outcome data. Loss to follow-up is mentioned and comparable.  **Low risk** | Data was gathered by primary health care facility staff, who also functioned as research assistants.  **Low risk** | Yes.  **Low risk** | Successful randomisation. Socio-economic status and obstetric confounding variables taken into account.  **Low risk** |
| 2013  Ross et al. | Odd numbers assigned to the control group and even numbers to the intervention group.  **High risk** | Order of approach.  **High risk** | No blinding, but outcome measurement not likely to be influenced by lack of blinding.  **Low risk** | Small population and a non-random component in sequence generation process.  **High risk** | No missing outcome data. No loss to follow-up.  **Low risk** | Data was gathered by registered nurses. Depressive symptoms were assessed using the 20-time Center for Epidemiological Studies Depression scale.  **Low risk** | Yes.  **Low risk** | Despite non-random component, demographic results did not differ.  **Low risk** |
| 2013  Tahir and  Al-Sadat | List of random codes was generated. Generation of group assignments used a blocked randomization method.  **Low risk** | Participants were included before randomization.  **Low risk** | Blinding assured.  **Low risk** | Participants are representative of the target population: mothers giving birth at a public hospital.  **Low risk** | No missing outcome data.  Loss to follow-up mentioned and comparable.  **Low risk** | Data was collected using a self-administered questionnaire and telephone interview by research team.  **Low risk** | Yes.  **Low risk** | Successful randomisation, infant age was controlled for in the statistical analyses.  **Low risk** |
| 2008  Jareethum et al. | Random allocation using a table of random numbers.  **Low risk** | Not clearly stated.  **Unclear risk** | Not clearly stated.  **Unclear risk** | Randomized.  **Low risk** | No missing outcome data.  Loss to follow-up mentioned and comparable.  **Low risk** | Data was gathered using a tested questionnaire. Pregnancy outcomes were collected from the obstetric records at the postpartum ward.  **Low risk** | Yes.  **Low risk** | Article does not state that confounders were taken into account.  **Unclear risk** |
| **Non-randomized study design** | | | | | | | | |
| 2014  Datta et al. | No control group, no randomization involved.  **High risk** | No control group, as such no randomization and need for allocation concealment.  **High risk** | No control group, so no blinding involved.  **High risk** | The respondents were selected using a multi-stage sampling technique.  **Low risk** | Not clearly stated.  **Unclear risk** | Data collected by the investigator.  **Low risk** | Yes.  **Low risk** | Not clearly stated.  **Unclear risk** |
| 2014  Jalloh-Vos et al. | Non-randomised step-wedge approach with an ‘internal’ non-intervention (counterfactual) group to compare interventions. Wedge allocation of chiefdoms was not done randomly, but by PHU density  **High risk** | Non-randomised step-wedge approach with an ‘internal’ non-intervention (counterfactual) group to compare interventions, unknown whether allocation was concealed until moment of assignment into wedges  **Unclear risk** | No blinding, but outcome measurement not likely to be influenced by lack of blinding.  **Low risk** | The individuals selected to participate are representative of the target population, though areas with no mobile phone reception and areas with few health care centres were excluded.  **Low risk** | No missing outcome data.  **Low risk** | Quantitative data was gathered using a questionnaire, survey, reports from health centres and data derived from information systems by data collectors who were trained prior to data collection.  **Low risk** | Yes.  **Low risk** | No regression analyses in which confounders were taken into account in quantitative analyses. Only descriptive (Chi, ttest, ANOVA). Information about baseline characteristics of two wedges is limited; which does not allow for assessment whether clusters were comparable or whether it would have been appropriate to consider confounders  **Unclear risk** |
| 2014  Oyeyemi and Wynn | Retrospective intervention study with a control group.  **High risk** | Retrospective intervention study with a control group.  **High risk** | Retrospective study.  **Low risk** | Case definition is adequate. Control area was selected on the basis of similar socio-economic and healthcare arrangements.  **Low risk** | Non-response rate was similar.  **Low risk** | Data collected from database.  **High risk** | Yes.  **Low risk** | Not clearly stated.  **Unclear risk** |
| 2013  Watkins et al. | Intervention area and control area were preselected.  **High risk** | Insufficient information to permit judgment of ‘Low risk’ or ‘High risk’.  **Unclear risk** | No blinding or incomplete blinding, but the review authors judge that the outcome is not likely to be influenced by lack of blinding.  **Low risk** | Investigators describe a random component in the sequence generation process, however, significant baseline differences existed between the intervention arms, intervention group and control group.  **Unclear risk** | No missing outcome data.  **Low risk** | Assessment of outcome occurred through self-report.  **High risk** | Yes.  **Low risk** | Article only states that confounders were taken into account, but no specific confounders are given.  **Unclear risk** |
| 2012  Pathak | Insufficient information to permit judgment.  **Unclear risk** | Insufficient information to permit judgment.  **Unclear risk** | The study did not address this outcome.  **Unclear risk** | Insufficient information about the selection process to permit judgment Yes or No.  **Unclear risk** | Insufficient reporting of attrition/exclusions to permit judgement of ‘Low risk’ or ‘High risk’.  **Unclear risk** | Self-measurements or data was gathered by adequate personnel.  **Low risk** | Yes.  **Low risk** | Article does not state that confounders are taken into account.  **Unclear risk** |
| 2010  Kaewkungwal et al. | No control group, no randomization involved.  **High risk** | No control group, as such no randomization and need for allocation concealment.  **High risk** | No control group, so no blinding involved.  **High risk** | All pregnant women and children of the area visiting the clinic were included.  **Low risk** | No missing outcome data.  **Low risk** | Data collected from databases.  **High risk** | Yes.  **Low risk** | Confounders defined and taken into account.  **Low risk** |
